# Our Philadelphia Letter

**Published:** 1886-11

**Authors:** Harry Huzza

**Affiliations:** Philadelphia


### Correspondence.

### OUR PHILADELPHIA LETTER.

THE MEDICAL COLLEGES OF PHILADELPHIA—A NEW ANTISEPTIC—PENNSYLVANIA'S ANATOMICAL ACT—ITS WORKING FULLY EXPLAINED, ETC.

*Editors Atlanta Medical and Surgical Journal:*

One more month has been added to the year, and a busy, eventful month it always proves. With the advent of fall and cool weather all things move briskly. We feel very perceptibly here that "Winter is i'coming in," yet it seems only to stimulate to more work.

Never have the medical colleges here been in better condition than at present. All auspices point to a studious, productive session. Jefferson still retains her popularity and prominence, as over five hundred matriculates amply show. The benches are crowded, and not only so, but with earnest, steady students, who evince by look and bearing they are here to work. The University of Pennsylvania has her full quota likewise and maintains her reputation. At the Woman's College the 37th annual session was to have been opened on October 7th by Dr. W. W. Keen, Professor of Surgery. His sudden though not serious illness disappointed the large audience which assembled to hear his address. Dean Rachel Bodley announced that the number of matriculates was larger than ever before, and that the college was never more prosperous. So 1886–87 will doubtless furnish many M. D.'s of future prominence.

The introductory lecture at Jefferson College was delivered, on the evening of September 30th, by Professor William F. Forbes, the new incumbent of the chair of anatomy. His subject was, "The Ideal Physician and his Character." In it he vividly pictured the great need for a high standard of attainment and conduct to be possessed by the medical profession ; the necessity for liberal culture to broaden the mind, to deepen the appreciation, to strengthen the devotion to truth, which is essential to true excellence ; showing also how all these things had been pointed out, even as early as Hippocrates, whose famous oath was but the embodiment of such principles. The address was most timely and interesting.

At the clinic there has been much that was instructive, but they must be seen to be appreciated. While no "startling" therapeutic agent has been sprung upon the world, still we have the newest antiseptic as an aspirant for honors. This is trichlorphenol, which is of Russian introduction, and has been favorably mentioned by one of the most prominent therapeutists. Trichlorphenol is extemporaneously prepared by mixing one part of a four *per cent.* solution of carbolic acid with five parts of a saturated solution of chlorinated lime ; the filtering is said to be twenty-five times more powerful than carbolic acid. It is certainly a good combination, and doubtless will prove useful. It has been chiefly employed, freely applied locally, in epidemic erysipelas. Its future is of course only to be foretold as yet by some medical "Wiggins."

The address of Dr. Forbes, upon making his advent into his new position in the college, naturally brings to mind some events in Jefferson's history with which he was prominently connected. A few students are here now who can remember the December day on which the daily papers came out in great headlines on "body-snatching," illustrated with maps and diagrams of the route from Lebanon Cemetery to the college, when policemen had to guard the buildings on account of the excited negroes, and students were not safe about Lombard and South streets. The publicity and excitement had much to do with awakening a better and proper sentiment, and shaping the present "anatomical act," by which dissection was legalized and provided for by law in Pennsylvania. A great era for medical education this was, placing it on a higher basis, and making instruction more thorough, careful and productive of good results than ever before. I have seen a number of references to the subject of an anatomical law in your able journal; as it is as great a need in Georgia now as it was formerly in Pennsylvania, I thought it might be of interest to send you a few points on the workings of the act here.

The "anatomical act," as it is called, provides for a board composed of the professors of anatomy and surgery at the different colleges. This board works chiefly through the executive committee, which is charged with carrying out the provisions of the law. In conversation with Dr. J. Ewing Mears, the demonstrator of surgery at Jefferson, and treasurer of the board, one of Philadelphia's most distinguished surgeons, I asked him about the anatomical law and its practical details. He said:

"The law works very efficiently and smoothly in Pennsylvania. The committee meets once a month, and assigns bodies to the different colleges in proportion to the number of students. We have a man hired who goes to the alms-house and the hospitals in the city, and conveys the bodies to the places to which they are assigned. This man is under contract, and returns his bill at the end of each month specifying the exact number thus carried. We also have a man who attends to the general dead, not from these institutions. Outside of the city, say in any one county, we arrange with the guardians of the poor, or the directors of the alms-house, to send the bodies coming under the provisions of the act. Regular boxes with special locks are provided, which we keep at these places and have an arrangement with the express companies by which they receive and forward them. So the superintendent of the alms-house, or whoever has the duty, delivers the body to the express company, who pay the charges of delivery, if there are any, and forward the box with the bill of expenses to us. It is directed to the undertaker in Philadelphia, who is our agent, and by him is delivered immediately to Jefferson College, from which point the proper distribution is made. At first it was difficult to get the express companies to undertake this contract, as they feared prominence and unpleasant talk. But everything has gone on smoothly and evenly. The bodies are delivered quickly and expeditiously without remark. In shipping we use a long oblong box, which is securely locked and fastened and does not attract attention.

"In carrying out this law, we do not force its construction too rigidly. We yield when there is opposition or feeling, are careful not to demand bodies in cases where, though there be no 'blood relations,' as the law provides, yet they are claimed, as say by a priest, by an employer of a servant, or in case of a person who left a dying request to be buried. We endeavor to let the law act smoothly without jarring—do not enforce it with an iron hand because it is law. Many instances could be given where we have been troubled, but patience with due consideration has always managed the matter. We take care not to 'strain' the law."

"How about the expenses incurred—who defrays them?"

"The fund for the expenses of the law's operation comes by assessment. The colleges are assessed in proportion to the number of students. For instance, in Jefferson, where we have about 400 students in the surgical and dissecting rooms, we assess, say 75 cents apiece; so with the other colleges. This money stays in the hands of the treasurer and goes to pay the necessary outlay."

"How do you provide against possible loss or theft of the bodies?"

"The agent who collects our bodies in the city receives duplicate receipts just as when a body is buried. One of these he retains and turns over with his bill at the end of each month. The other is left with the janitor of the college, to which the body is carried, and goes to the secretary of the board with the monthly report. Thus both the receipts go on file, serving to check each other and the agent's bill. Besides, every body is accompanied by a death certificate which is deposited with the Board of Health. These are checked off once a month by our secretary, providing another safeguard against error. These means prevent any trouble or danger of a dishonest agent who might sell the body or otherwise dispose of it. Now a person outside, or our agent himself, might bring as many bodies as he pleased, but he could not dispose of them or smuggle them in, as he would not have proper receipts nor death certificates 'from a reputable physician.' I have had applications occasionally from different portions of the State to know if I would take a body, but I always look upon them with suspicion. I have replied simply that the law does not permit 'the traffic in bodies.' We only receive those that come to us in the regular way and only pay for carrying them. So likewise I have inquiries from other States about selling bodies, but of course that is contrary to law. The bodies can only be used 'within this State' for *scientific purposes*, and each college is under bond to that effect."

"How are you notified in cases where death occurs outside a public institution and the body is unclaimed ?"

"Where any one dies in a private house and there is no one to bury the body, application is always made to the proper officials for a coffin. It is thus brought to the attention of the authorities and is sent to the alms-house, from which place it is conveyed by our agent just as those that die there. So it is the coroner's duty to turn over to us all *unclaimed* bodies he may have at any time in his charge. Likewise those from the morgue and prisons must be duly reported and delivered. Every safeguard is taken that the law shall be properly carried out. The bodies are virtually buried, and all precautions are used, just as in ordinary interment, that there shall have been no wrong cause of death, nor anything done out of the proper way. This is all attended to entirely free of expense to the State, county or city. Everything is carefully regulated, and no loop-hole is left unguarded, so that it is duly by bond 'conditioned that all such bodies which the said physician, or surgeon, or the said school or college shall receive thereafter shall be used only for the promotion of medical science within this State.' "

Such are a few points about the anatomical law and its workings as given by one of the leading officers of the board. Of its good effects, the thorough, practical, systematic instruction in surgery and anatomy given by the Philadelphia schools is sufficient evidence. It provides for a need, an indispensable need, an absolute requirement in the study of medicine, for anatomy is the foundation of all medical knowledge, and it provides for it in the quickest, the fairest and least objectionable way. The workings of the Pennsylvania law have demonstrated its justice and its utility. Yours truly,

*Philadelphia, Oct. 20th, 1886.* Harry Huzza.

